# Dipeptidyl Peptidase-4 Inhibitor-Associated Bullous Pemphigoid in an Elderly Woman With Type 2 Diabetes Mellitus: A Case Report

**DOI:** 10.7759/cureus.110686

**Published:** 2026-06-11

**Authors:** Guillermo Roa Alvarez, Mario Shuchleib Cukiert, Felix R Hernández Altamirano, Cristina Berumen Glinz, Maria E Vega Memije

**Affiliations:** 1 School of Medicine and Health Sciences, Tecnológico de Monterrey, Mexico City, MEX; 2 Department of Dermatology, Hospital General Dr. Manuel Gea Gonzalez, Mexico City, MEX; 3 Department of Dermatopathology, Hospital General Dr. Manuel Gea Gonzalez, Mexico City, MEX

**Keywords:** bullous pemphigoid, diabetes mellitus, dpp-4 inhibitor, dpp-4 inhibitor–associated bullous pemphigoid, drug-induced autoimmune blistering disease, linagliptin

## Abstract

Bullous pemphigoid (BP) is the most common autoimmune subepidermal blistering disease and predominantly affects older adults. In recent years, dipeptidyl peptidase-4 inhibitors (DPP-4i), widely prescribed for type 2 diabetes mellitus, have emerged as important pharmacologic triggers of BP. DPP-4 inhibitors as a class have been associated with BP, with the strongest evidence reported for vildagliptin, although cases involving linagliptin have also been documented. Linagliptin has also been increasingly recognized as a potential trigger of drug-associated disease. We report the case of an 81-year-old woman with long-standing type 2 diabetes mellitus treated with metformin and linagliptin who developed a generalized pruritic vesiculobullous eruption. Dermatologic examination demonstrated multiple tense bullae, erosions, crusted plaques, and post-inflammatory hyperpigmented lesions involving the trunk, extremities, and intertriginous regions. Histopathologic examination revealed a subepidermal blister with prominent eosinophilic infiltration. Direct immunofluorescence demonstrated linear C3 and IgG deposition along the basement membrane zone, while indirect immunofluorescence localized immunoreactants to the roof of the split, confirming the diagnosis of BP. The temporal association with linagliptin exposure and the absence of alternative triggers supported the diagnosis of DPP-4 inhibitor-associated BP. Linagliptin was discontinued, and treatment with prednisone was initiated, resulting in progressive improvement and complete cessation of new blister formation at follow-up. This case highlights the importance of recognizing medication-induced BP in older diabetic patients and reviews current evidence regarding the epidemiology, pathogenesis, clinical presentation, diagnosis, and management of DPP-4 inhibitor-associated BP.

## Introduction

Bullous pemphigoid (BP) is the most common autoimmune subepidermal blistering disorder, characterized by autoantibodies directed against the hemidesmosomal proteins BP180 (collagen XVII) and BP230, located within the dermoepidermal junction [1]. The disease primarily affects elderly individuals and typically presents with intense pruritus, urticarial plaques, and tense bullae distributed over the trunk and extremities [1,2].

The incidence of BP has increased worldwide over the last two decades, a phenomenon partly attributed to population aging and increasing exposure to medications capable of inducing autoimmunity [3]. Among these medications, dipeptidyl peptidase-4 inhibitors (DPP-4i), also known as gliptins, have emerged as one of the strongest pharmacologic risk factors for BP development [4-8].

Multiple epidemiologic studies from Europe and Asia have demonstrated a significantly increased risk of BP among diabetic patients receiving DPP-4 inhibitors, with reported odds ratios ranging from approximately 2 to 4 compared with non-users [5-8]. Although vildagliptin appears to confer the highest risk, increasing evidence implicates linagliptin, sitagliptin, and other agents in the class [4-8].

We report a case of generalized BP associated with linagliptin exposure in an elderly woman and discuss the current literature regarding DPP-4 inhibitor-associated BP.

## Case presentation

An 81-year-old woman from Puebla, Mexico, residing in Mexico City, presented with a several-month history of progressive pruritic blistering skin lesions. Her medical history was significant for long-standing type 2 diabetes mellitus treated with metformin and linagliptin, as well as hypertension treated with losartan. Her previous surgical history included cholecystectomy, inguinal hernia repair, bilateral cataract extraction with intraocular lens implantation, and hysterectomy. Clinical information obtained during dermatology evaluation revealed disease onset in November 2023. The patient first developed generalized pruritus, followed by erythematous plaques and subsequently tense bullae over the ensuing weeks. Previous treatments included topical betamethasone, cetirizine, oral acyclovir, and Domeboro soaks, without significant improvement. No recent medication changes other than chronic linagliptin exposure were identified. There was no history of neurologic disease, active malignancy, recent infection, radiotherapy, or other recognized BP triggers.

**Figure 1:**
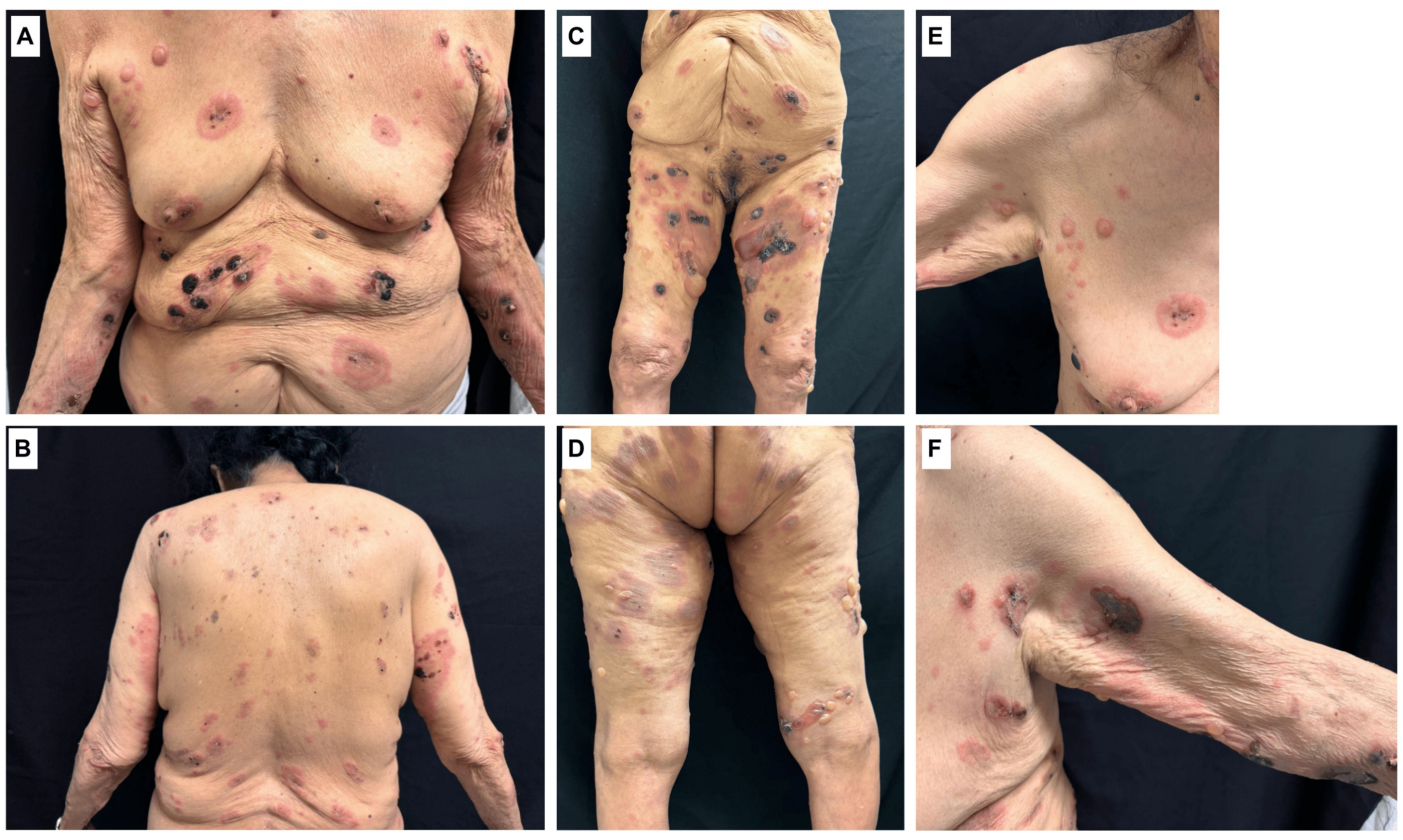
Clinical presentation of DPP-4 inhibitor-associated bullous pemphigoid. (A) Frontal view demonstrating multiple tense bullae, erosions, hemorrhagic crusts, and annular erythematous plaques involving the chest, abdomen, and upper extremities. (B) Posterior view showing scattered erosions, crusted lesions, and post-inflammatory hyperpigmented macules distributed across the back and upper arms. (C) Anterior lower-body view revealing numerous tense bullae and crusted plaques affecting the thighs and inguinal region. (D) Posterior lower-body view demonstrating bilateral involvement of the gluteal regions and posterior thighs, with active bullae, erosions, and residual hyperpigmentation. (E) Close-up of the left axillary region showing multiple intact tense bullae arising on an erythematous base. (F) Right axillary detail demonstrating hemorrhagic crusted erosions, intact bullae, and surrounding inflammatory plaques. Clinical findings are consistent with a generalized inflammatory phenotype of DPP-4 inhibitor-associated bullous pemphigoid. DPP-4: Dipeptidyl peptidase-4.

Physical examination demonstrated a generalized vesiculobullous eruption characterized by multiple tense serous bullae, erythematous plaques, erosions, hemorrhagic crusts, and post-inflammatory hyperpigmented macules involving the trunk, upper extremities, lower extremities, axillary regions, inframammary folds, and inguinal areas (Figure 1). Several intact bullae measuring approximately 0.5-2 cm in diameter were present on erythematous bases, while numerous lesions had evolved into erosions with overlying crust formation. Extensive residual hyperpigmentation suggested recurrent episodes of blister formation and healing. The oral, ocular, genital, and nasal mucosae were examined and showed no involvement. Clinical morphology was highly suggestive of a generalized autoimmune subepidermal blistering disorder.

**Figure 2:**
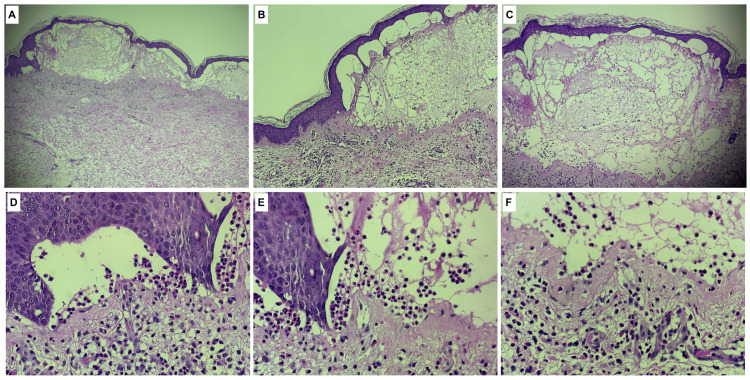
Histopathologic examination demonstrating a subepidermal blister with eosinophil-rich inflammation (H&E stain). (A) Low-power view (H&E, ×40) showing a large subepidermal blister cavity beneath an intact epidermis, with an associated superficial dermal inflammatory infiltrate. (B) Intermediate-power view (H&E, ×100) demonstrating separation at the dermoepidermal junction with a multiloculated subepidermal blister containing inflammatory cells. (C) Intermediate-power view (H&E, ×100) highlighting an extensive subepidermal blister cavity with numerous eosinophils dispersed throughout the blister lumen. (D) High-power view (H&E, ×400) showing abundant eosinophils within the blister cavity and papillary dermis. (E) High-power view (H&E, ×400) demonstrating eosinophilic spongiosis and eosinophil migration toward the dermoepidermal junction. (F) High-power view (H&E, ×400) revealing a dense superficial perivascular and interstitial eosinophil-rich inflammatory infiltrate. These findings are consistent with bullous pemphigoid.

A lesional skin biopsy was obtained for routine histopathologic examination. Microscopic evaluation demonstrated a subepidermal blister containing numerous inflammatory cells, predominantly eosinophils. The superficial dermis showed a dense eosinophil-rich inflammatory infiltrate extending along the dermoepidermal junction. The absence of acantholysis argued against pemphigus vulgaris, while eosinophil-rich subepidermal blistering favored bullous pemphigoid. These findings were consistent with bullous pemphigoid (Figure 2).

Linagliptin was discontinued following the diagnosis. The patient was treated with clobetasol propionate 0.05% cream twice daily and oral prednisone at an initial dose of 50 mg/day, followed by gradual tapering. Progressive clinical improvement was observed over the subsequent months, with cessation of new blister formation and resolution of pruritus. At the 12-month follow-up visit, no new bullae were noted, and only residual post-inflammatory hyperpigmentation remained.

Given the patient’s age, characteristic clinicopathologic findings, history of prolonged exposure to linagliptin, and the growing recognition of DPP-4 inhibitors as causative agents in BP, a diagnosis of linagliptin-associated bullous pemphigoid was established.

## Discussion

Bullous pemphigoid is a chronic autoimmune blistering disease mediated by autoantibodies directed against BP180 and BP230, structural proteins essential for epidermal adhesion [1]. Binding of these autoantibodies activates complement, recruits inflammatory cells, particularly eosinophils, and ultimately produces dermoepidermal separation, resulting in tense blister formation [1,2].

During the past decade, DPP-4 inhibitors have become increasingly recognized as important triggers of BP. Initial pharmacovigilance reports were followed by larger case-control and population-based studies confirming a significant association between DPP-4 inhibitor exposure and BP development [4-8]. Benzaquen M et al. demonstrated that DPP-4 inhibitor use was independently associated with BP in a multicenter case-control study from France and Switzerland [5]. Similar findings were subsequently reported in Finnish, Japanese, Korean, and Israeli cohorts [6-8].

Although the exact pathogenic mechanism remains incompletely understood, several hypotheses have been proposed. DPP-4 (CD26) is expressed on keratinocytes, T lymphocytes, eosinophils, and numerous other immune cells. Inhibition of DPP-4 may alter immune regulation, enhance eosinophilic inflammation, and modify antigen processing at the basement membrane zone [9]. Experimental studies suggest that DPP-4 inhibition interferes with plasmin-mediated cleavage of BP180, potentially exposing neoepitopes capable of triggering autoantibody formation [9,10]. This mechanism may explain the distinctive serologic profiles observed in DPP-4 inhibitor-associated BP.

Interestingly, DPP-4 inhibitor-associated BP may differ from conventional BP. Several studies have identified a non-inflammatory phenotype characterized by fewer erythematous lesions and reduced reactivity against the NC16A domain of BP180 [11,12]. Nevertheless, inflammatory phenotypes resembling classic BP have also been reported, particularly among Western populations [11,12]. Unlike the non-inflammatory phenotype characterized by limited erythema reported in some DPP-4 inhibitor-associated BP cohorts, our patient exhibited an inflammatory phenotype with widespread erythematous plaques, extensive tense bullae, erosions, and eosinophil-rich histopathology.

Genetic susceptibility may also contribute to disease development. Ujiie H et al. identified HLA-DQB1*03:01 as a potential genetic marker associated with DPP-4 inhibitor-induced BP in Japanese patients [13]. However, additional studies are required to clarify the relevance of this association across diverse populations.

Recognition of medication-induced BP is clinically important because withdrawal of the offending drug frequently contributes to disease control. Several reports have documented improvement following discontinuation of the implicated DPP-4 inhibitor, although many patients still require corticosteroids and adjunctive immunosuppressive therapy [4,10]. Current treatment recommendations generally mirror those for conventional BP and include high-potency topical corticosteroids, systemic corticosteroids, doxycycline, immunosuppressive agents, and increasingly biologic therapies in refractory disease [14].

The present case illustrates several classic features of DPP-4 inhibitor-associated BP: advanced age, diabetes mellitus, prolonged exposure to a gliptin, generalized tense bullae, eosinophil-rich subepidermal blistering, and confirmatory immunofluorescence findings. Given the widespread use of DPP-4 inhibitors among elderly diabetic patients, dermatologists, internists, and endocrinologists should maintain a high index of suspicion when evaluating new-onset blistering eruptions.

Applying the Naranjo Adverse Drug Reaction Probability Scale yielded a score of +2, corresponding to a probable adverse drug reaction. Although rechallenge was not performed for ethical reasons, the temporal relationship between drug exposure, disease onset, and subsequent clinical improvement following drug withdrawal supports a probable association.

Limitations include the absence of BP180/BP230 ELISA testing and the lack of rechallenge, which precludes definitive establishment of causality. Nevertheless, the temporal relationship, clinicopathologic findings, exclusion of alternative triggers, and favorable response following linagliptin withdrawal and treatment support a probable association.

## Conclusions

DPP-4 inhibitor-associated bullous pemphigoid represents an increasingly recognized form of drug-induced autoimmunity. Linagliptin should be considered a potential causative agent in elderly diabetic patients presenting with generalized pruritic blistering eruptions. Histopathology and immunofluorescence remain essential for diagnosis, while careful medication review is critical for identifying potential triggers. Early recognition and discontinuation of the offending agent may facilitate disease control and improve patient outcomes.

## References

[1] Murrell DF, Daniel BS, Joly P (2012). Definitions and outcome measures for bullous pemphigoid: recommendations by an international panel of experts. J Am Acad Dermatol.

[2] Kobayashi M, Amagai M, Kuroda-Kinoshita K, Hashimoto T, Shirakata Y, Hashimoto K (2002). BP180 ELISA using bacterial recombinant NC16A protein as a diagnostic and monitoring tool for bullous pemphigoid. J Dermatol Sci.

[3] Marazza G, Pham HC, Schärer L (2009). Incidence of bullous pemphigoid and pemphigus in Switzerland: a 2-year prospective study. Br J Dermatol.

[4] Béné J, Moulis G, Bennani I (2016). Bullous pemphigoid and dipeptidyl peptidase IV inhibitors: a case-noncase study in the French Pharmacovigilance Database. Br J Dermatol.

[5] Benzaquen M, Borradori L, Berbis P, Cazzaniga S, Valero R, Richard MA, Feldmeyer L (2018). Dipeptidyl peptidase IV inhibitors, a risk factor for bullous pemphigoid: retrospective multicenter case-control study from France and Switzerland. J Am Acad Dermatol.

[6] Varpuluoma O, Försti AK, Jokelainen J, Turpeinen M, Timonen M, Huilaja L, Tasanen K (2018). Vildagliptin significantly increases the risk of bullous pemphigoid: a Finnish nationwide registry study. J Invest Dermatol.

[7] Kridin K, Bergman R (2018). Association of bullous pemphigoid with dipeptidyl-peptidase 4 inhibitors in patients with diabetes: estimating the risk of the new agents and characterizing the patients. JAMA Dermatol.

[8] Lee SG, Lee HJ, Yoon MS, Kim DH (2019). Association of dipeptidyl peptidase 4 inhibitor use with risk of bullous pemphigoid in patients with diabetes. JAMA Dermatol.

[9] Takama H, Yoshida M, Izumi K (2018). Dipeptidyl peptidase-4 inhibitor-associated bullous pemphigoid: recurrence with epitope spreading. Acta Derm Venereol.

[10] Lindgren O, Varpuluoma O, Tuusa J, Ilonen J, Huilaja L, Kokkonen N, Tasanen K (2019). Gliptin-associated bullous pemphigoid and the expression of dipeptidyl peptidase-4/CD26 in bullous pemphigoid. Acta Derm Venereol.

[11] Izumi K, Nishie W, Mai Y (2016). Autoantibody profile differentiates between inflammatory and noninflammatory bullous pemphigoid. J Invest Dermatol.

[12] Horikawa H, Kurihara Y, Funakoshi T (2018). Unique clinical and serological features of bullous pemphigoid associated with dipeptidyl peptidase-4 inhibitors. Br J Dermatol.

[13] Ujiie H, Muramatsu K, Mushiroda T (2018). HLA-DQB1*03:01 as a biomarker for genetic susceptibility to bullous pemphigoid induced by DPP-4 inhibitors. J Invest Dermatol.

[14] Feliciani C, Joly P, Jonkman MF (2015). Management of bullous pemphigoid: the European Dermatology Forum consensus in collaboration with the European Academy of Dermatology and Venereology. Br J Dermatol.

